# Spontaneous closure of a chronic full-thickness idiopathic macular hole after Irvine-Gass syndrome resolution

**DOI:** 10.1186/s12886-022-02354-6

**Published:** 2022-03-24

**Authors:** Douglas Rodrigues da Costa, Pedro Gomes Oliveira Braga, Leonardo Eleuterio Ariello, Leandro Cabral Zacharias

**Affiliations:** Department of Ophthalmology, Hospital das Clínicas, University of São Paulo Medical School, São Paulo, Brazil

**Keywords:** Idiopathic macular hole, Irvine Gass syndrome, Pseudophakic macular edema

## Abstract

**Background:**

Full-thickness idiopathic macular hole (IMH) usually causes serious visual deformities and visual acuity loss. Pseudophakic cystic macular edema, also known as Irvine-Gass syndrome, is another entity that causes visual disturbances, and occurs mainly after cataract extraction. We present a case report of a patient that was diagnosed with a full-thickness macular hole that spontaneously closed after the resolution of an Irvine-Gass syndrome, which occurred after an uneventful cataract extraction.

**Case presentation:**

A 75 years-old female presented with the complaints of decreased visual acuity and color contrast sensitivity on both eyes (OU) and central visual field deformations on her left eye (LE). She was diagnosed with a full-thickness IMH on her LE, and cataract on OU. After an uneventful cataract extraction via phacoemulsification, she developed an Irvine-Gass syndrome at her LE, which was treated topically. The IMH closed spontaneously after the resolution of the Irvine-Gass syndrome, and the patient is being followed with no further complaints.

**Conclusion:**

The exact mechanism for spontaneous closure of full-thickness idiopathic macular holes is still not completely understood. In this case, we hypothesize that the coalesced intraretinal cysts caused by the Irvine-Gass syndrome formed a bridge-like structure connecting the inner walls of the macular hole, thus connecting the remnants of the Muller cells which enabled the full recovery of the normal foveal structure.

## Background

Idiopathic full-thickness macular hole (IMH) causes serious central visual field loss, visual deformation and decrease in visual acuity. Despite that the main treatment is surgical, mainly through internal limiting membrane peeling via pars plana vitrectomy, there are sporadic cases in which spontaneous closure is documented [1–4]. Irvine-Gass syndrome (pseudophakic cystoid macular edema) is another entity observed after cataract extraction, that may cause visual acuity loss [5]. Most of the cases resolve spontaneously, and the first line of treatment in persistent cases consist of topical use of corticosteroids and nonsteroidal anti-inflammatory drugs, usually with great response and visual acuity improvement [6].

This case report presents a patient with an idiopathic full-thickness macular hole with spontaneous closure after Irvine-Gass syndrome resolution that occurred after an uneventful cataract extraction via phacoemulsification.

## Case presentation

In February 2021, a 75-year-old female patient presented with the complaint of progressive visual loss, low color sensitivity on both eyes (OU) and central metamorphopsia on the left eye (LE) over the last 4 years. She had a medical history of asthma with weekly use of inhalant corticosteroids. Despite the long-term complaints, she did not have any previous ophthalmologic examinations due to socio-economic issues.

**Fig. 1:**
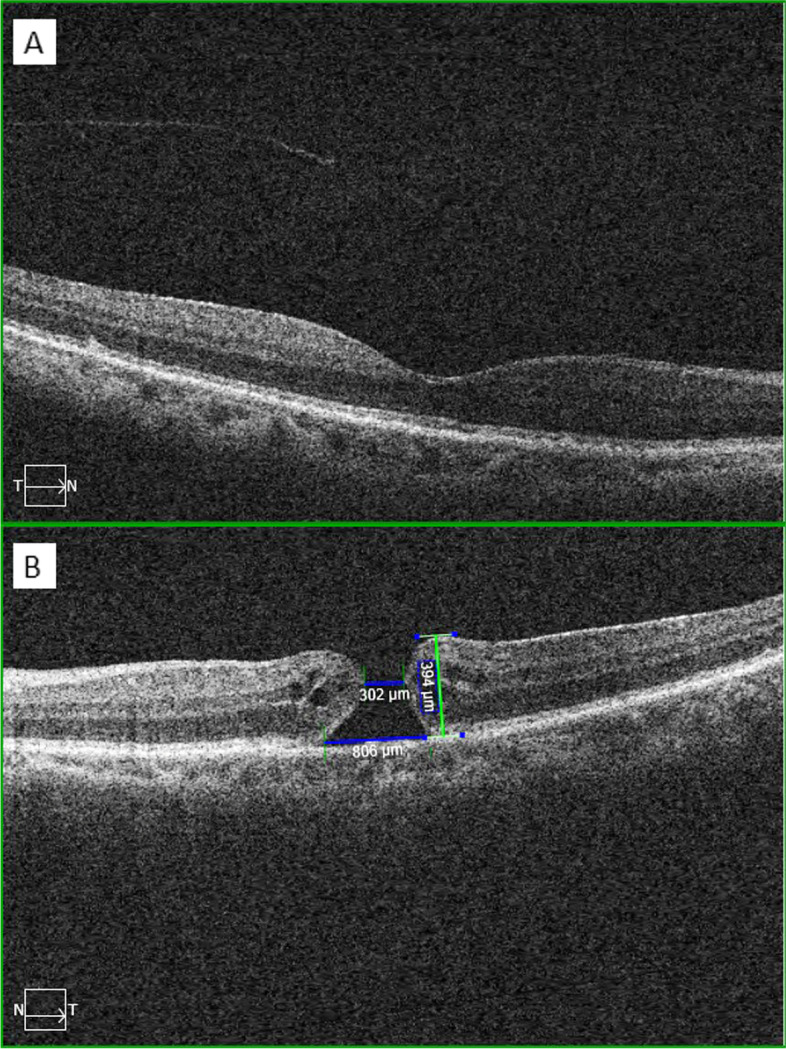
Both eyes optical coherence tomography image of the macula taken by the Zeiss Cirrus 6000 (Zeiss International, Germany) device. **A** Right eye macula presents with normal foveal depression and partial vitreous detachment; **B** Left eye macula presents a full thickness macular hole with an inner diameter of 302 µm, outer diameter of 806 µm and height of 394 µm. Small intraretinal cysts could be noted and there were no vitreous-macular traction components

Her best corrected visual acuity was 20/40 on her RE and 20/200 on her LE. On slit-lamp examination, cataract was present on OU, both classified as grade 2 nuclear associated with posterior subcapsular grade 1 outside the visual axis. Her intraocular pressure was 14 mmHg OU, and both ocular motility and pupillary reflexes were unremarkable. Ophthalmoscopy of the RE was also unremarkable, while on the left eye, a macular hole was observed, with positive Watzke-Allen sign. To complement the clinical examination, a swept-source optical coherence tomographic (SS-OCT) was performed. The right eye examination showed normal foveal depression and partial vitreous detachment (Fig. 1A), and on LE there was a full-thickness macular hole, classified as stage 2 according to the Gass classification [7] (Fig. 1B).

**Fig. 2:**
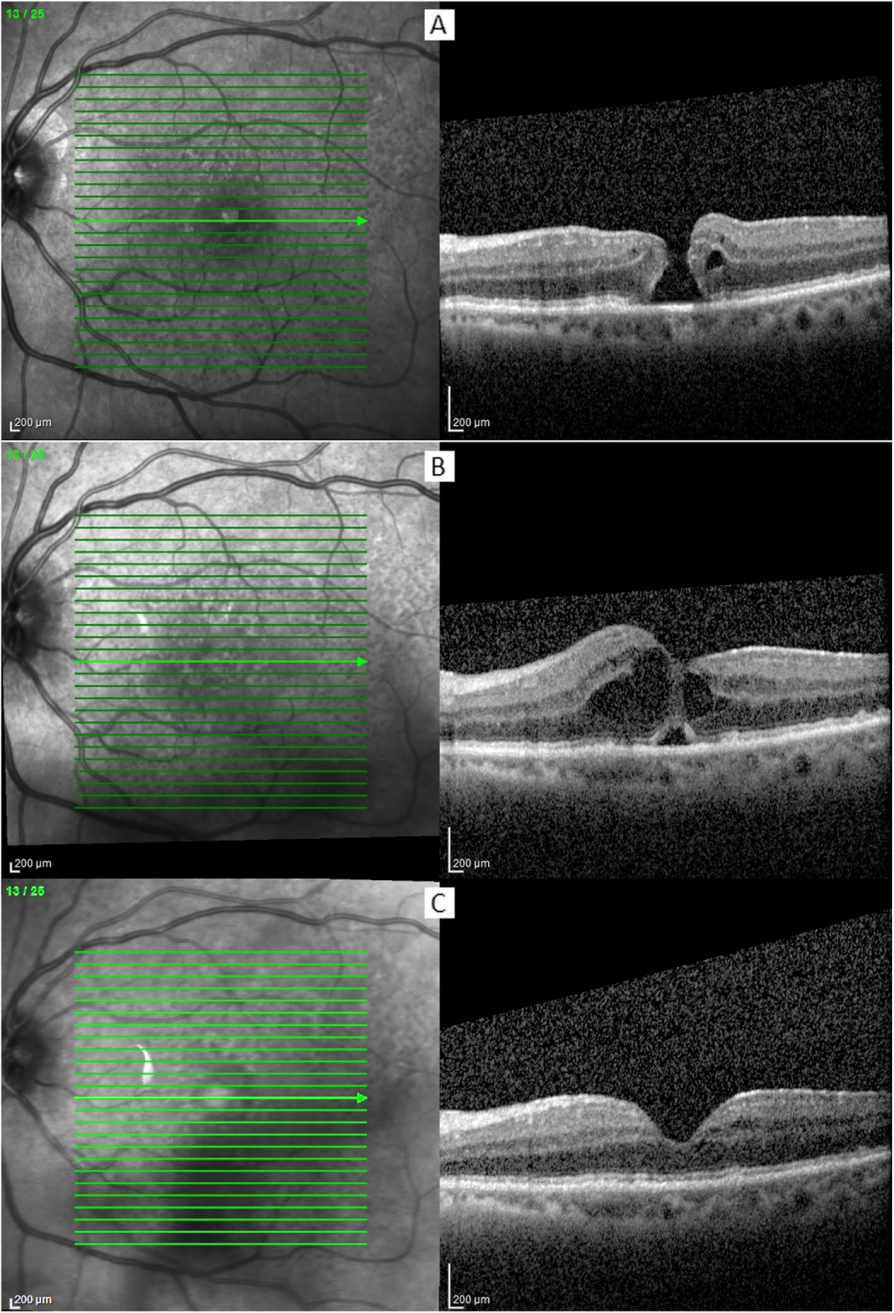
Evolution of the full-thickness macular hole of the left eye, documented by the Spectralis OCT (Heidelberg, Germany). The images were acquired in a different device due to maintenance of the Zeiss Cirrus 6000 device and the horizontal cuts were performed on the inferior edge of the macular hole; **A** After four months of the initial diagnose, the full-thickness macular hole diameter remained stable, with the presence of intraretinal cysts; **B** Irvine-Gass syndrome was diagnosed due to the presence of coalesced intraretinal cysts, which formed a bridge-like structure at the level of the inner plexiform layer; **C** After the resolution of the Irvine-Gass syndrome, normal foveal depression and full recovery of the outer retinal layers could be noted

Initially, it was decided to follow the patient without vitreoretinal surgery, since the duration of the IMH was not clear. After four months, the patient returned to ophthalmologic examination and the IMH’s size on the LE remained unchanged (Fig. 2A). The patient refused to be submitted to a posterior vitrectomy via pars plana, however she agreed on proceeding with cataract extraction via phacoemulsification and intraocular lens implantation on the LE as an attempt to improve her visual acuity. The surgery was performed on June 2021, 4 months after the IMH was diagnosed, and occurred with no complications. On the first post-operative evaluation (1^st^ PO), her visual acuity on LE was 20/100 and the remain of the ophthalmological examination was unremarkable. On the 7^th^ PO the examination remained the same, however, on the 30^rd^ PO she complained of decreased visual acuity on her LE. Her visual acuity dropped to 20/400, cornea was transparent, the intraocular lens was well placed, and on ophthalmoscopy, macular cysts could be noted. The SS-OCT demonstrated retinal thickening, hyporeflective cystic areas within the macula and enlargement of the retinal nerve fiber layer (Fig. 2B). Therefore, the diagnosis of Irvine-Gass syndrome was made, and clinical treatment was promptly initiated with topical nonsteroidal anti-inflammatory drugs, 3 times a day.

Since the patient presented socio-economic issues, she refused to return once every week and her next appointment was set to 30 days after the diagnosis of the Irvine-Gass syndrome. She returned referring that all her LE’s complaints were completely resolved, including the central visual field metamorphopsias. Her best corrected visual acuity on LE was 20/25; slit lamp examination was unremarkable and on ophthalmoscopy the foveal depression could be noted. Her SS-OCT showed resolution of the Irvine-Gass syndrome and complete closure of the full-thickness macular hole with full recovery of the external retinal layers (Fig. 2C). The patient was then submitted to an uneventful cataract extraction on her RE and is being followed since with no further complaints.

## Discussion and conclusion

Pseudophakic cystic edema, frequently referred as to Irvine-Gass syndrome, is the main cause of decrease in visual acuity after cataract extraction with or without implantation of intraocular lenses [8]. The incidence of Irvine-Gass syndrome is highly variable via modern phacoemulsification, with studies varying from 4 to 40%, since there are several subclinical undiagnosed cases [9]. Its incidence is higher in intracapsular extraction, followed by extracapsular extraction and phacoemulsification [10]. It mainly occurs after complicated surgeries, with rupture of the posterior capsule and vitreous loss [9]. However, it may occur at lower rates even after uneventful surgeries.

Irvine-Gass syndrome physiopathology is multifactorial, and it lies mostly on inflammation due to surgical manipulation. Blood-aqueous and blood-retinal barriers are broken by several cytokines related to the inflammatory cascade, which leads to increased vascular permeability [11]. Even after uneventful surgeries, the paracentral macular area may be thickened, specially the superior, temporal and nasal quadrants [12]. If the increased vascular permeability exceeds the retinal pigmented epithelium capacity of drainage, microcysts formed in the outer plexiform and inner nuclear layers of the retina coalesces into cysts, leading to the Irvine-Gass syndrome [8].

Irvine-Gass syndrome mainly resolves spontaneously, but eventually it might be necessary to be treated with topical corticosteroids, nonsteroidal anti-inflammatories and, in some cases, intravitreal drugs such as corticosteroids or anti-vascular endothelial growth factors (anti-VEGF) [9]. Pars plana vitrectomy is an option if Irvine-Gass syndrome is complicated by vitreoretinal traction or is chronically unresponsive to medical treatment [13].

Full-thickness macular holes are formed after the foveola’s disruption of the inner Muller cell layer [14]. The retina is structurally supported by microtubules and intermediate filaments in Muller cells and adherent junctions between Muller cells and astrocytes [15]. The fovea contains only one type of microglia, which is the Muller glia and astrocytes in the perifovea [15, 16]. Muller cells provide mechanical forces to resist to the stretch resulting from anteroposterior or tangential tractional forces that occurs, for example, after partial detachment of the posterior vitreous and in cases of cystoid macular edema. Also, it is presumed that these cells are involved in the restoration of the foveal shape after resolution of full-thickness macular holes, in spontaneous closure or surgically treated cases [17].

A previous review study has shown that certain characteristics are more associated to spontaneous closure of full-thickness idiopathic macular holes [18]. Spontaneous closure of IMH may happen in about 6% of the cases [19]. Usually, it occurs after 3 to 4 months from the initial diagnosis and is more common when the hole has less than 400 µm in diameter, especially less than 250 µm**.** The authors also suggest that some OCT findings are more suggestive of spontaneous closure, such as the relieve of vitreous macular traction, formation of a bridge-like structure at the borders of the macular hole, epiretinal membrane and cystic structure [20]. In our case, the IMH had an initial diameter of 302 µm, no vitreous macular traction was observed, and there was presence of small intraretinal cysts. After the cataract extraction, multiple intraretinal cysts were formed and coalesced due to the Irvine-Gass syndrome. We hypothesize that the formation of theses cysts and enlargement of the retinal layers induced the formation of a bridge-like structure that connected the inner walls of the hole at the level of the outer plexiform layer (Fig. 2B).

Bringmann et al.proposed that spontaneous closure of full-thickness macular holes happens through the fusion of the remnants of the Muller cell cone and the Muller cell structures at the external limiting membrane [14, 16]. This regeneration is mediated by a centripetal contraction of Muller cell side processes at the level of the outer plexiform layer. The normal fovea is then formed by an increase in thickness of the outer nuclear layer which is produced by Muller cells. Afterwards, at the end of this process, the central photoreceptors segments are regenerated. In the current case, the normal fovea shape and layers presented full recover after the resolution of the Irvine-Gass syndrome (Fig. 2C).

To the best of our knowledge, this is the first case report of an idiopathic full-thickness macular hole that closed spontaneously after the treatment and resolution of an Irvine-Gass syndrome. There are several reports of spontaneous closure of IMH, including those formed as a complication after Irvine-Gass syndrome [1, 20]. Despite the exact mechanism that enable spontaneous closure of full-thickness IMH, we support the presumed role of the Muller cells, since the coalesced intraretinal cysts formed by the Irvine-Gass syndrome allowed a connection between the Muller cells of the inner walls of the macular hole and consequent regeneration of the normal foveal structure.

## Data Availability

All photos and patient’s data are available (contact Correspondent author for data and material).

## Abbreviations

IMH: Idiopathic macular hole
RE: Right eye
LE: Left eye
OU: Oculus uterque (both eyes)
PO: Post-operative
SS-OCT: Swept-source optical coherence tomographic
anti-VEGF: Anti-vascular endothelial growth factors
